# Beyond the Pump: The Evolving Molecular Landscape of Intrahepatic Cholestasis

**DOI:** 10.3390/diagnostics16050726

**Published:** 2026-02-28

**Authors:** Ilaria Ziccardi, Michela Zorzi, Adamo Pio d’Adamo

**Affiliations:** 1 Institute for Maternal and Child Health—IRCCS “Burlo Garofolo”, 34137 Trieste, Italy; ilaria.ziccardi@burlo.trieste.it (I.Z.); michela.zorzi@burlo.trieste.it (M.Z.); 2 Department of Medicine, Surgery and Health Sciences, University of Trieste, 34129 Trieste, Italy

**Keywords:** cholestasis, progressive familial intrahepatic cholestasis (PFIC), genetics, Next-Generation Sequencing (NGS), precision medicine, IBAT inhibitors, bile acids

## Abstract

Cholestasis encompasses a broad spectrum of hepatobiliary disorders characterized by impaired bile formation or flow. Historically classified based on clinical onset and severity, the landscape of cholestatic liver disease has been revolutionized by the advent of high-throughput genomic technologies. This review elucidates the critical role of genetics in redefining the pathophysiology, diagnosis, and management of cholestasis, framing pediatric Progressive Familial Intrahepatic Cholestasis (PFIC) and Adult-Onset Cholestatic Disease (AOCD) as a continuous phenotypic spectrum. We discuss the expansion of the molecular nosology to include 13 distinct PFIC types, highlighting how defects in canalicular transporters, tight junctions, and nuclear receptors underpin clinical heterogeneity. Furthermore, we examine the paradigm shift in the diagnostic flowchart, where Next-Generation Sequencing (NGS) has largely superseded liver biopsy for etiological definition. Finally, we address the therapeutic implications of this molecular precision, demonstrating how specific genotypes dictate eligibility for novel targeted therapies, such as IBAT inhibitors, marking the transition from supportive care to personalized medicine.

## 1. Introduction: Pathophysiology and Clinical Spectrum

The term cholestasis derives from the Greek *cholé* (bile) and *stasis* (stagnation) and describes a pathophysiological state characterized by impaired bile formation and/or flow, resulting in the retention of potentially toxic bile acids and other biliary components within the liver and systemic circulation. At the cellular level, retained hydrophobic bile acids exert direct hepatotoxicity through multiple mechanisms, including impairment of mitochondrial electron transport chain (ETC) complexes, disruption of mitochondrial dynamics (fission/fusion balance), and induction of oxidative stress via the mitochondrial permeability transition pore, ultimately promoting hepatocyte apoptosis and inflammatory signaling [1,2]. Cholestasis is classified as intrahepatic or extrahepatic depending on the site of impairment and is defined as chronic when symptoms persist for more than six months [3].

Chronic cholestatic conditions are predominantly intrahepatic in origin, arising from hepatocellular functional defects (functional cholestasis) or lesions affecting the microscopic intrahepatic biliary tree. Conversely, extrahepatic (or obstructive) cholestasis is caused by mechanical obstruction of the large bile ducts outside the liver, such as in sclerosing cholangitis or biliary lithiasis [3,4].

Patients present with a wide spectrum of clinical signs, among which pruritus, jaundice, fatigue, acholic stools, dark urine, and malabsorption of dietary lipids and fat-soluble vitamins represent the most distinctive features. The etiology of cholestasis is diverse, encompassing viral hepatitis, total parenteral nutrition (TPN)-associated cholestasis, drug-induced liver injury (DILI), mechanical obstructions (e.g., gallstones, tumors), and genetic disorders. Among these, genetic defects provide a unique molecular window into the fundamental mechanisms of bile formation and enterohepatic circulation and serve as the primary focus of this review [3,5].

**Figure 1:**
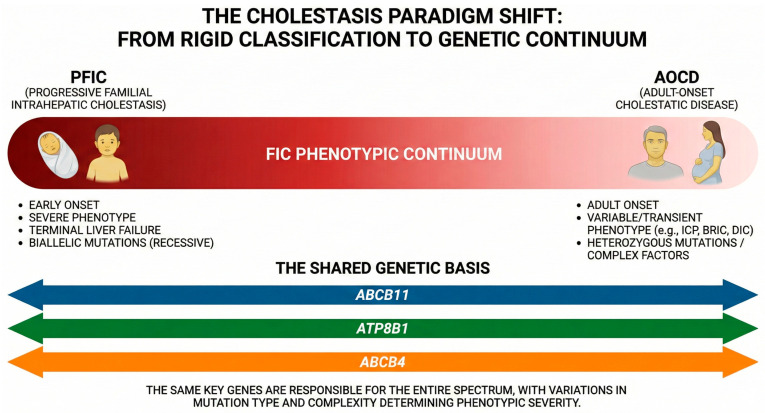
The phenotypic continuum of Familial Intrahepatic Cholestasis (FIC). This diagram illustrates the paradigm shift from a rigid, age-based classification to a unified molecular spectrum. (**Left**) Pediatric Progressive Familial Intrahepatic Cholestasis (PFIC) represents the severe, early-onset end of the spectrum, typically driven by high-penetrance biallelic mutations and progressing to terminal liver failure. (**Right**) Adult-Onset Cholestatic Disease (AOCD) occupies the milder end, characterized by variable or transient phenotypes such as Intrahepatic Cholestasis of Pregnancy (ICP), Benign Recurrent Intrahepatic Cholestasis (BRIC), and Drug-Induced Cholestasis (DIC), often associated with heterozygous variants and environmental triggers. The arrows highlight the shared genetic basis, demonstrating that the same key transporter genes (*ABCB11*, *ATP8B1*, *ABCB4*) underpin the entire spectrum, with clinical severity determined by the specific mutational burden and complexity.

Cholestatic disorders affect both pediatric and adult populations, displaying a phenotypic continuum with distinct clinical trajectories (Figure 1). While pediatric forms typically manifest with severe, chronic presentations, adult-onset phenotypes are generally milder and frequently intermittent and transient. However, the genetic underpinnings of these conditions often overlap, establishing significant pathophysiological links between pediatric and adult cholestatic phenotypes [6,7,8].

## 2. The Genetic Basis of Pediatric Cholestasis

Among hereditary cholestatic conditions, Progressive Familial Intrahepatic Cholestasis (PFIC) constitutes the most well-defined group. It is characterized by pediatric onset and a severe phenotype—including jaundice, intractable pruritus, hepatosplenomegaly, and growth failure due to malabsorption—often progressing to liver failure. Clinical symptoms typically arise in the neonatal period or within the first year of life, advancing to end-stage liver disease (ESLD) between infancy and adolescence. Ultimately, the condition requires liver transplantation or leads to death, depending on the severity of the presentation [9,10].

**Figure 2:**
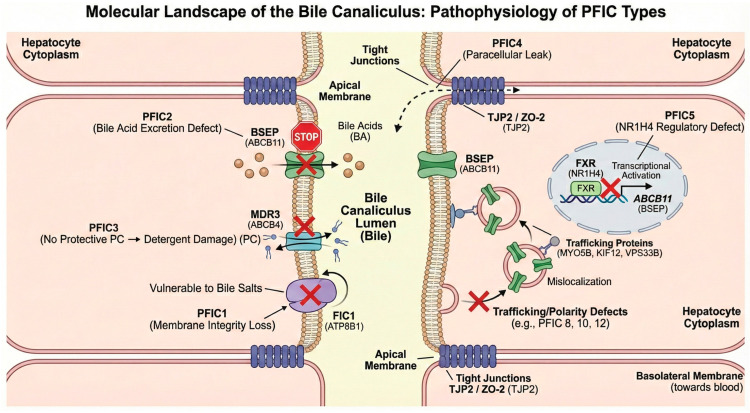
The molecular landscape of the bile canaliculus and pathophysiology of PFIC subtypes. This scheme illustrates the diverse molecular mechanisms underlying cholestasis at the hepatocyte apical membrane. (**Left**) The “classical” forms involve direct defects in canalicular transporters and membrane dynamics: PFIC2 (*ABCB11*) results in failed bile acid excretion, PFIC3 (*ABCB4*) impairs phosphatidylcholine secretion leading to bile salt-induced membrane damage, and PFIC1 (*ATP8B1*) compromises membrane lipid asymmetry. (**Right**) Newer subtypes reveal complex non-transporter mechanisms: PFIC4 (*TJP2*) disrupts tight junction integrity, causing paracellular bile leakage; PFIC5 (*NR1H4*) represents a nuclear regulatory defect where FXR failure prevents the transcriptional upregulation of *ABCB11*. Additionally, trafficking defects (e.g., PFIC8, 10, 12) involve motor and fusion proteins (KIF12, MYO5B, VPS33B) that cause the intracellular mislocalization of otherwise functional transporters.

PFIC encompasses a spectrum of distinct disorders following an autosomal recessive inheritance pattern. Historically, the nosology was anchored to clinical phenotype and biochemical features. The advent of high-throughput sequencing has redefined this landscape. Currently, OMIM recognizes 13 distinct types of PFIC, each driven by mutations in a specific gene [8]. This genetic heterogeneity reflects a broader pathophysiological reality: cholestasis can arise not only from direct pump failure but also from defects in tight junction integrity, vesicle trafficking, and nuclear receptor signaling (Figure 2). While the “classical” forms (PFIC1–3) remain the most prevalent, this expanded classification (summarized in Table 1) highlights the intricate complexity of the adaptive signaling pathways governing hepatobiliary homeostasis [11].

### 2.1. Classical Transporter Defects (PFIC1, 2, 3)

PFIC2 is the most common subtype, followed by PFIC3, while PFIC1 is relatively rare. These “classical” forms are associated with genes that are also implicated in transient or milder cholestatic conditions in adults, creating a genetic bridge across the age spectrum.

PFIC1 is caused by biallelic mutations affecting the P-type ATPase FIC1, encoded by the *ATP8B1* gene (OMIM 211600). FIC1 maintains the asymmetric distribution of phospholipids in the canalicular membrane, thereby protecting the hepatocyte from bile salt-induced damage. This function complements that of MDR3, which protects the biliary epithelium by secreting phosphatidylcholine into bile. Loss-of-function mutations render the membrane vulnerable to bile salt-induced damage [12,13]. Pathogenic variants in *ATP8B1* are also associated with milder forms such as Benign Recurrent Intrahepatic Cholestasis-1 (BRIC1) and Intrahepatic Cholestasis of Pregnancy-1 (ICP1) [14].

PFIC2 results from biallelic mutations in the *ABCB11* gene (OMIM 601847), encoding the bile salt export pump (BSEP). Mutations lead to defective BSEP expression or function, impairing the excretion of conjugated bile acids. A hallmark feature is the significant intrahepatic retention of bile salts with normal or low serum gamma-glutamyl transferase (GGT) levels, as the low biliary bile acid concentration is insufficient to damage the biliary epithelium and release GGT [3]. *ABCB11* mutations are also found in BRIC2 and increase susceptibility to ICP [14].

PFIC3 is caused by biallelic mutations in the *ABCB4* gene (OMIM 602347), encoding the MDR3 floppase, which translocates phosphatidylcholine (PC) into bile. Unlike types 1 and 2, PFIC3 is characterized by elevated serum GGT levels, resulting from the detergent effect of free bile salts on cholangiocytes in the absence of protective phospholipids [15]. *ABCB4* variants are associated with a broad spectrum of adult phenotypes, including biliary fibrosis, cirrhosis, and ICP [14,16].

Notably, the relevance of *ABCB4* deficiency in biliary pathology is further underscored by the Mdr2 (*Abcb4*) knockout mouse, the murine orthologue of human MDR3/*ABCB4*, which spontaneously develops biliary fibrosis with features resembling primary sclerosing cholangitis, thus providing a valuable preclinical model for studying the progression from phospholipid secretion failure to sclerosing cholangiopathy [17].

### 2.2. Defects in Canalicular Integrity and Regulation (PFIC4, 5)

Recent advances have identified PFIC subtypes caused by mechanisms beyond direct transporter failure, involving tight junction integrity and nuclear receptor signaling.

PFIC4 (OMIM 615878) results from biallelic mutations in the *TJP2* gene. The encoded protein (TJP2/ZO-2) is crucial for the assembly of tight junctions. The hypothesized mechanism involves the failure of claudin-1 to localize to the bile canaliculi seals, leading to paracellular leakage of bile acids into the liver parenchyma. This creates a specific hepatic microenvironment where constant exposure to bile acids causes severe damage, explaining why the liver is the primary site of pathology despite the protein’s ubiquitous expression [18,19].

PFIC5 (OMIM 617049) is caused by mutations in the *NR1H4* gene (encoding FXR), the master transcriptional regulator of bile acid homeostasis. This represents a regulatory defect: the absence of FXR prevents the upregulation of BSEP and other transporters [20]. Clinically, PFIC5 presents with normal GGT and a distinctive early-onset vitamin K-independent coagulopathy, reflecting FXR’s role in regulating coagulation factors [21]. Histology mimics PFIC2 (absent BSEP), but the primary defect is upstream at the transcriptional level [8,22].

### 2.3. Emerging Subtypes: Intracellular Trafficking and Cell Polarity (PFIC6–13)

The most recent expansion of the PFIC spectrum underscores that a functional transporter is ineffective if it cannot effectively reach the canalicular membrane. This group comprises disorders caused by defects in intracellular trafficking, cell polarity, and structural scaffolding. Genes such as *MYO5B* (PFIC10) and *KIF12* (PFIC8) encode motor proteins, while *VPS33B* (PFIC12) encodes a Sec1/Munc18-family protein that regulates vesicle fusion. Together, these are essential for the polarization of the hepatocyte and the vesicular transport of apical proteins [23,24,25,26,27]. Mutations in these genes result in the “mislocalization” of BSEP and MDR3, mimicking a primary transporter defect despite the transporter genes themselves being intact. Additionally, this category includes defects in the basolateral export machinery (*SLC51A*, PFIC6, OMIM 619484) [28,29] and other junctional or signaling components (*USP53*, PFIC7, OMIM 619658; *SEMA7A*, PFIC11, *ZFYVE19*, PFIC9), further illustrating that cholestasis can originate from dysfunction at any step of the bile secretion machinery [30,31,32,33].

## 3. Adult-Onset Cholestatic Disease (AOCD) and the Clinical Continuum

Adult-Onset Cholestatic Disease (AOCD) encompasses a heterogeneous group of conditions characterized by variable severity, including Benign Recurrent Intrahepatic Cholestasis (BRIC), Intrahepatic Cholestasis of Pregnancy (ICP), Drug-Induced Cholestasis (DIC), and Low-Phospholipid-Associated Cholelithiasis (LPAC). Unlike the Mendelian recessive patterns of pediatric forms, AOCD is often complex, involving heterozygous variants, incomplete penetrance, and environmental triggers.

Benign Recurrent Intrahepatic Cholestasis (BRIC) is characterized by recurrent, self-limiting episodes of jaundice and severe pruritus separated by symptom-free intervals [34]. While it shares genetic etiologies with PFIC (*ATP8B1*, *ABCB11*), BRIC can present in both biallelic and monoallelic configurations [3,35,36].

Intrahepatic Cholestasis of Pregnancy (ICP) manifests as reversible cholestasis in the second or third trimester. It represents a paradigm of gene-environment interaction, where the hormonal load of pregnancy unmasks a genetic predisposition (e.g., heterozygous variants in *ABCB4*, *ATP8B1*, *ABCB11*) [14,37,38,39].

Drug-Induced Cholestasis (DIC) highlights the role of pharmacogenetics. About 15–20% of the population are low expressers of ABC transporters [40], creating a susceptibility background. Drugs may inhibit BSEP (e.g., cyclosporine, rifampicin) or MRP2 [41,42,43,44,45,46]. Genetic variants in these transporters significantly increase the risk of Drug-Induced Liver Injury (DILI), transforming an idiosyncratic reaction into a predictable vulnerability [47,48,49,50].

Low-Phospholipid-Associated Cholelithiasis (LPAC) is a genetic disorder caused by *ABCB4* variants leading to impaired biliary phospholipid secretion, resulting in highly lithogenic bile and recurrent intrahepatic and gallbladder cholesterol cholelithiasis. LPAC typically presents in young adults (before age 40) and is characterized by recurrence of biliary symptoms after cholecystectomy and intrahepatic hyperechogenic foci on ultrasound. *ABCB4* variants are identified in approximately 50% of cases and are mostly heterozygous [51,52,53]. LPAC further expands the spectrum of *ABCB4*-related adult liver disease, alongside ICP and biliary fibrosis, and its chronic evolution may lead to secondary sclerosing cholangitis, biliary cirrhosis, or, rarely, cholangiocarcinoma [53,54].

### The Unified Spectrum

The integration of high-throughput genetics with clinical phenotyping has revolutionized the classification of these disorders. The boundaries between pediatric and adult presentations have become indistinct, framing these conditions as a unified Familial Intrahepatic Cholestasis (FIC) continuum. PFIC subtypes represent the severe, early-onset extreme of this spectrum, while AOCD occupies the milder, variable end. Over the years, it has become evident that the same genes responsible for severe PFIC are frequently associated with adult-onset manifestations [7,35,55]. Consequently, AOCD typically manifests as chronic, manageable conditions or transient cholestatic episodes that resolve without significant sequelae, although in some cases they may progress rapidly to liver fibrosis or malignancy [8].

## 4. Diagnosis of Cholestasis

**Figure 3:**
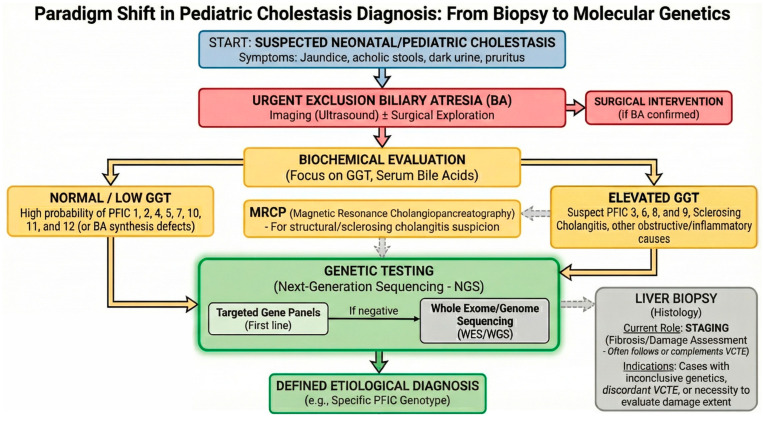
The evolved diagnostic algorithm for pediatric cholestasis: from histology to molecular precision. This flowchart illustrates the paradigm shift from a biopsy-centered approach to a “genotype-first” strategy in the evaluation of cholestatic infants. The diagnostic pathway begins with the initial clinical suspicion based on presenting symptoms (jaundice, acholic stools, dark urine, pruritus) (**Blue**), followed by the mandatory and urgent exclusion of biliary atresia (BA) through imaging and, when necessary, surgical exploration (**Red**). Biochemical profiling serves as the initial phenotypic discriminator (**Yellow**): serum gamma-glutamyl transferase (GGT) levels act as a “phenotypic compass”, where normal or low GGT directs suspicion toward PFIC types 1, 2, 4, 5, 7, 10, 11, and 12 (or bile acid synthesis defects), while elevated GGT suggests PFIC3, 6, 8, and 9, sclerosing cholangitis, or other obstructive/inflammatory etiologies. Magnetic resonance cholangiopancreatography (MRCP) is indicated when structural or sclerosing cholangiopathy is suspected. Next-Generation Sequencing (NGS) has largely superseded liver biopsy for etiological definition (**Green**): the strategy prioritizes targeted gene panels as the first-line approach, escalating to Whole Exome/Genome Sequencing (WES/WGS) if initial screening is negative, ultimately leading to a defined etiological diagnosis. Liver biopsy and non-invasive fibrosis assessment by vibration-controlled transient elastography (VCTE) (**Gray**) are reserved for staging hepatic fibrosis, for cases with inconclusive or discordant genetic results, or when evaluation of tissue damage extent is clinically required. Dashed arrows indicate non-routine, conditional, or complementary diagnostic steps. In this modern framework, liver biopsy is no longer the primary diagnostic tool but a complementary modality to genetic testing.

The accurate diagnosis of cholestatic liver diseases mandates a comprehensive, multidisciplinary approach integrating clinical evaluation, biochemical analysis, radiological imaging, histological examination, and, increasingly, genetic testing [56] (Figure 3). A detailed patient history serves as the foundation of this process, providing the essential context to guide subsequent investigations [3].

A tailored diagnostic strategy taking age-specific pathophysiology into account is imperative. In the pediatric population, the primary clinical objective is the rapid exclusion of biliary atresia (BA), which accounts for 25–40% of obstructive jaundice cases in the first three months of life and requires urgent surgical intervention [57]. Once surgical causes are excluded, the differential diagnosis broadens to include a spectrum of metabolic and genetic defects. This includes structural bile duct defects, congenital hormonal deficiencies, immune-mediated liver disorders, drug-induced cholestasis, and specific genetic conditions presenting later in childhood [58].

### 4.1. Role of ESPGHAN Guidelines in Pediatric Practice

While the European Association for the Study of the Liver (EASL) guidelines provide comprehensive recommendations for the management of cholestatic liver diseases across all age groups [36], the European Society for Pediatric Gastroenterology, Hepatology and Nutrition (ESPGHAN) offers specific guidance tailored to the pediatric population [57,59]. ESPGHAN recommendations emphasize the urgency of diagnostic workup in neonatal cholestasis, the importance of early referral to specialized pediatric hepatology centers, and age-appropriate nutritional interventions. The integration of both EASL and ESPGHAN guidelines ensures optimal care across the entire age spectrum, from neonates to adults transitioning from pediatric services.

### 4.2. Biochemical Investigations: The Phenotypic Compass

Since hyperbilirubinemia and impaired bile acid flux are clinical hallmarks of cholestasis, current guidelines designate serum markers—including direct bilirubin, aminotransferases (ALT/AST), alkaline phosphatase (ALP), gamma-glutamyl transferase (GGT), and markers of synthetic function (glucose, albumin, coagulative profile)—as the first-line investigation to define etiology and severity [57].

Among these, GGT levels represent a pivotal diagnostic discriminator, specifically guiding the genetic suspicion. While GGT and ALP are typically elevated in most pediatric cholestatic conditions [60], a specific subset of hereditary disorders—namely PFIC types 1, 2, 4, 5, 7, 10, 11, and 12—paradoxically present with normal or low GGT levels relative to the degree of cholestasis [56,61]. Recognizing this “low GGT” signature is crucial for directing subsequent molecular testing.

Regarding diagnostic thresholds, both the EASL and the Chinese Medical Association recommend using cut-offs of 1.5 times the upper limit of normal (ULN) for ALP and 3 times the ULN for GGT [3,62]. Moreover, given that biochemical sensitivity varies and the true diagnostic value of these parameters can be uncertain, these markers should be interpreted within the broader clinical context [56]. Additionally, testing for antinuclear (ANA) and antimitochondrial (AMA) antibodies is recommended to exclude autoimmune etiologies, particularly when an overlap between primary sclerosing cholangitis and autoimmune hepatitis is suspected [3,56].

### 4.3. Imaging and Histological Evaluation

In conjunction with biochemical screening, non-invasive imaging remains the initial step for both pediatric and adult patients. Abdominal ultrasound is primarily used to rule out extrahepatic obstruction [63], while advanced modalities such as computed tomography (CT) and magnetic resonance cholangiopancreatography (MRCP) aid in further distinguishing intrahepatic from extrahepatic cholestasis [56].

Historically, liver biopsy has been considered the gold standard for the definitive diagnosis of intrahepatic cholestasis in 90–95% of cases [63,64,65]. However, liver biopsy is an invasive procedure carrying inherent risks. Consequently, in the current era of precision medicine, there is a paradigm shift towards prioritizing non-invasive molecular investigations. While histology remains indispensable for staging liver fibrosis or when laboratory results are inconclusive [56], Vibration-Controlled Transient Elastography (VCTE) has emerged as a validated non-invasive tool for the assessment of liver fibrosis, offering a reliable surrogate for histological staging in both pediatric and adult cholestatic populations and reducing the need for repeated biopsies during longitudinal follow-up [66]. The integration of Next-Generation Sequencing (NGS) increasingly allows for an accurate etiological diagnosis without the immediate need for invasive sampling. The two approaches are now complementary: biopsy assesses the extent of damage (staging)—increasingly supported or preceded by VCTE as a non-invasive alternative—while genetics defines the cause (etiology) [36,56,66].

### 4.4. Role of Genetic Testing

The advent of molecular diagnostics has revolutionized the management of cholestasis. The most recent EASL Clinical Practice Guidelines on genetic cholestatic liver diseases strongly recommend genetic testing for infants and children with unexplained cholestasis, as well as for adults after excluding common causes, particularly in cases with atypical features or resistance to standard therapy [36].

#### 4.4.1. Diagnostic Strategy: From Panels to Whole Exome Sequencing

Genetic investigation is paramount in early-onset disease, where high-penetrance variants often drive the phenotype. While targeted gene panels have traditionally been the first-line approach [67], they present a static limitation: they can only detect what is already known. A significant proportion of patients—including pediatric cases with a clear intrahepatic cholestasis phenotype—remains genetically undiagnosed using standard approaches: in a recent single-center pediatric cohort, WES identified disease-causing variants in only 31% of 166 children with intrahepatic cholestasis, and re-analysis of previously negative data led to additional diagnoses through newly published genes [68,69]. This diagnostic gap strongly suggests the existence of causative genes not yet linked to cholestasis in current databases. Consequently, from a prospective standpoint, Whole Exome Sequencing (WES) and Whole Genome Sequencing (WGS) represent superior diagnostic tools. Unlike restricted panels, WES and WGS offer an unbiased genomic interrogation, essential for identifying novel cholestatic genes in “orphan” cases. Furthermore, advanced NGS pipelines now allow for the detection of Copy Number Variations (CNVs)—such as large deletions or duplications—which are frequently missed by standard sequencing but constitute a relevant fraction of pathogenic alleles in cholestasis. Therefore, in patients testing negative on panels, the escalation to WES/WGS is not merely a second step but a necessary strategy to uncover new disease mechanisms [36].

#### 4.4.2. The Challenge of Interpretation: VUS and Functional Validation

The broad application of WES/WGS introduces a new challenge: the interpretation of Variants of Uncertain Significance (VUS). The existence of a phenotypic continuum complicates the establishment of clear genotype–phenotype correlations. Because the same genes can drive diverse clinical outcomes depending on the specific mutational impact, distinguishing pathogenic drivers from benign bystanders is challenging. While initial classification often relies on computational predictions, these are frequently insufficient for the nuanced defects seen in the cholestatic spectrum.

Consequently, the American College of Medical Genetics and Genomics (ACMG) guidelines emphasize the need for evolving evidence to refine classification. Specifically, the PS3 (Pathogenic Strong) and BS3 (Benign Strong) criteria highlight the decisive value of well-established functional studies [70]. These criteria allow robust experimental data demonstrating a deleterious or neutral effect to carry strong evidentiary weight, which can provide the proof needed to reclassify a VUS. This underscores that accurate diagnosis along the FIC continuum relies not merely on sequencing, but on the rigorous integration of genetic data with clinical phenotyping and functional validation. For patients remaining undiagnosed, EASL guidelines recommend re-analyzing sequencing data every three years to capture novel disease-gene associations or reclassify VUS [36,56,66].

#### 4.4.3. Genotype–Phenotype Correlations and Clinical Prognostication

A notable feature of hereditary cholestatic disorders is the phenotypic variability observed among affected individuals. While intrafamilial variability suggests a role for modifier genes and environmental influences in modulating disease expression [71], recent large-scale multicenter evidence has established that the *ABCB11* genotype is the primary driver of clinical outcome in BSEP deficiency.

Data from the NAPPED (NAtural course and Prognosis of PFIC and Effect of biliary Diversion) consortium [72] have validated a risk stratification model classifying patients into three distinct groups: BSEP1 (carrying at least one common mild mutation: p.D482G or E297G) [72,73,74,75,76], BSEP2 (at least one missense variant different from BSEP1), and BSEP3 (biallelic protein-truncating mutations, leading to nonfunctional protein or absent BSEP expression). This classification correlates strictly with Native Liver Survival (NLS), which drops significantly from a median of 20.4 years in BSEP1 to just 3.5 years in BSEP3 patients [72].

Understanding these correlations has immediate clinical implications. Crucially, the genotype predicts not only severity but also therapeutic response: while surgical biliary diversion (SBD) effectively extends native liver survival in BSEP1 and BSEP2 patients, it provides no significant benefit in the BSEP3 group, who also face a markedly higher risk of hepatocellular carcinoma (34% by age 15). Consequently, phenotypic prediction is no longer just an active area of research but a validated clinical tool to guide surgical decision-making and transplant prioritization [72,77].

### 4.5. Diagnosis of Specific Cholestatic Disorders

Applying these diagnostic principles allows for the precise characterization of specific clinical entities within the cholestatic spectrum

#### 4.5.1. Benign Recurrent Intrahepatic Cholestasis (BRIC)

BRIC represents the milder, non-progressive end of the PFIC-BRIC continuum. It shares allelic etiology with severe forms (PFIC) primarily in genes such as *ATP8B1* (BRIC1; OMIM 243300) [78] and *ABCB11* (BRIC2; OMIM 605479) [79] but typically involves missense variants that retain some residual protein function [80].

Clinically, the diagnosis has traditionally followed established criteria, including multiple distinct episodes of jaundice, separated by asymptomatic periods lasting a minimum of six months, in addition to the exclusion of both medication-induced or toxin-related cholestasis and biliary tract pathology [81]. A suspicion of BRIC is raised when a first cholestatic episode resolves spontaneously, particularly in the presence of a positive family history. However, differential diagnosis is crucial and, to be definitive, typically requires the documentation of a second symptomatic episode. Essential exclusion criteria during the initial attack include acute or chronic viral hepatitis and drug-induced liver injury. Confirmatory features include the documentation of excess fecal bile acid excretion during asymptomatic intervals and normal liver histology months after the resolution of the attack [82]. Today, molecular analysis can expedite this diagnostic timeline, identifying the specific pathogenic variants and differentiating BRIC from progressive forms without the need to await a recurrent episode.

#### 4.5.2. Intrahepatic Cholestasis of Pregnancy (ICP)

ICP is diagnosed based on pruritus, elevated serum total bile acids and liver enzymes, the exclusion of alternative liver pathologies, and the spontaneous resolution of abnormalities approximately three months postpartum [38]. Clinically, ICP presents with varying severity of transaminase and bile acid elevation, with mild hyperbilirubinemia occurring in approximately 30% of cases [83,84]. From a genetic perspective, ICP is increasingly recognized as a manifestation of underlying genetic susceptibility “unmasked” by the hormonal stress of pregnancy. Heterozygous variants in ABC transporter genes (*ABCB4*, *ABCB11*, *ATP8B1*) and other cholestasis-associated loci significantly predispose women to ICP [14,85]. Identifying these variants is clinically critical not only for maternal management but for familial risk assessment: these variants may be inherited by offspring, potentially placing the child at risk for developing severe PFIC if inherited in a recessive pattern or as complex compound heterozygosity. The hormonal milieu of late pregnancy unmasks this genetic susceptibility through multiple converging mechanisms: 17β-estradiol transcriptionally represses BSEP via ERα-mediated interference with FXR signaling [86,87], while sulfated progesterone metabolites—particularly epiallopregnanolone sulfate—competitively inhibit FXR function, further reducing BSEP expression and bile acid efflux [88,89]. These endocrine mechanisms, reviewed in detail elsewhere [90], reinforce the gene-environment paradigm that characterizes ICP within the broader FIC continuum.

#### 4.5.3. Drug-Induced Cholestasis (DIC)

The diagnosis of DIC is primarily one of exclusion, requiring a meticulous medical history to rule out competing etiologies [91]. A detailed pharmacological anamnesis, covering drugs, herbs, and supplements taken in the preceding 6 months, is mandatory [92]. Diagnostic confidence is strengthened by observing worsening liver injury upon rechallenge and improvement following drug withdrawal [93]. Liver injury is biochemically classified as cholestatic when there is a >2-fold elevation in ALP or when the ALT/ALP ratio is ≤2. While DILI is largely idiosyncratic, the genetic component involves susceptibility factors rather than deterministic mutations (e.g., specific HLA alleles) [50]. Although routine screening for cholestatic gene variants is not currently mandated, research suggests that defects in canalicular transporters may lower the threshold for drug toxicity, highlighting the future potential of pharmacogenomics.

#### 4.5.4. Low-Phospholipid-Associated Cholelithiasis (LPAC)

LPAC syndrome should be suspected when at least two of the following criteria are met: onset of biliary symptoms before age 40, recurrence of biliary symptoms after cholecystectomy, and presence of intrahepatic hyperechogenic foci or microlithiasis on ultrasonography [52,54]. Additional features supporting the diagnosis include a personal history of ICP and a family history of gallstones in first-degree relatives [94]. Imaging—particularly liver ultrasound with color Doppler, CT, and MRCP—plays a central role in detecting intrahepatic stones, while *ABCB4* genotyping confirms the diagnosis, although variants are identified in only approximately half of cases [95,96]. Early recognition is critical, as LPAC management differs substantially from common gallstone disease: UDCA therapy [10 mg/kg/day] is effective in preventing stone formation and recurrence, and cholecystectomy alone is often insufficient without concomitant medical treatment [54].

## 5. Therapeutic Management: From Supportive Care to Precision Medicine

The precise molecular characterization achieved through the diagnostic flowchart described above is the prerequisite for selecting the most appropriate therapeutic strategy. Treatment approaches have evolved from purely symptomatic relief to genotype-guided interventions that target the specific molecular defect.

### 5.1. PFIC Management

Treatment for PFIC is multimodal. While medical therapy remains the first line for all types, its long-term impact on prognosis is variable.

Nutritional support constitutes a fundamental component of PFIC management. Dietary lipids are mainly provided as medium-chain triglycerides (MCTs), as they do not require bile salts for absorption, thereby improving the overall nutritional condition [3]. Supplementation of both water- and fat-soluble vitamins is generally required to correct deficiencies associated with chronic cholestasis.

The pharmacological approach is increasingly stratified by the patient’s specific genetic profile.

Ursodeoxycholic acid (UDCA), a non-hepatotoxic hydrophilic bile acid that modifies the composition of the endogenous bile acid pool [97], is effective for both pediatric and adult-onset forms [98]. However, response to UDCA is strongly genotype-dependent. Its efficacy relies on efficient hepatocellular uptake mediated by NTCP (*SLC10A1*) [99], and variations in this gene may influence intracellular bioavailability [100]. Regarding efflux defects, patients with PFIC3 (*ABCB4*) harboring missense mutations with residual phospholipid secretion typically respond well, whereas those with biallelic truncating mutations (complete absence of MDR3) often fail to respond [101]. A similar genotype-response correlation is observed in PFIC2. Symptomatic pruritus control is achieved with conventional agents, including rifampicin and cholestyramine.

Prior to the advent of IBAT inhibitors, bile acid sequestrants—principally cholestyramine—represented the main pharmacological approach to interrupt the enterohepatic circulation of bile acids for the symptomatic relief of cholestatic pruritus. Cholestyramine is a positively charged non-absorbable anion exchange resin that non-specifically binds bile acids in the intestinal lumen, preventing their reabsorption and promoting fecal excretion [102]. However, cholestyramine carries several significant limitations that are particularly relevant in the context of hereditary cholestasis. First, its mechanism is non-selective: the resin binds not only bile acids but also a wide range of co-administered drugs and nutrients, mandating that all concurrent medications—including UDCA and fat-soluble vitamin supplements—be taken at least one hour before or four to six hours after cholestyramine administration to avoid impaired absorption [103]. This is of particular concern in PFIC patients, who already suffer from malabsorption of fat-soluble vitamins as a direct consequence of cholestasis, and in whom cholestyramine can further compound these deficiencies [104]. Second, palatability is notoriously poor, as the granular powder requires suspension in liquid and produces a gritty, unpleasant oral sensation, leading to very low adherence rates, especially in the pediatric population [105,106]. Third, the evidence base for cholestyramine efficacy in PFIC is limited, relying primarily on historical clinical experience and retrospective data rather than randomized controlled trials [102]. Finally, cholestyramine has low efficacy in severe cholestatic diseases where bile acid secretion into the intestinal lumen is already markedly reduced, which is the case in the majority of severe pediatric cholestatic conditions [107].

In contrast, IBAT inhibitors represent a paradigm shift from non-specific intraluminal binding to targeted pharmacological blockade. By selectively and reversibly inhibiting the ileal bile acid transporter (*SLC10A2*), these agents achieve a more precise interruption of bile acid reabsorption at the molecular level, with a significantly more favorable drug-interaction profile and improved tolerability compared to bile acid sequestrants [102,108]. Crucially, unlike cholestyramine, IBAT inhibitors have been validated through rigorous Phase 3 randomized, placebo-controlled trials (PEDFIC-1, MARCH-PFIC), establishing an evidence-based therapeutic standard for PFIC [109,110].

IBAT inhibitors represent the first approved pharmacological treatments specifically for PFIC [111]. Odevixibat (Bylvay^®^) was approved by the EMA and FDA in 2021 [112] for the treatment of cholestatic pruritus in PFIC patients aged ≥3 months. In the PEDFIC-1 trial, odevixibat demonstrated significant reductions in serum bile acids and pruritus scores compared to placebo [109].

Maralixibat (Livmarli^®^), initially approved for Alagille syndrome [113], has subsequently received approval for PFIC, with the MARCH-PFIC trial demonstrating robust efficacy [110]. Recent indirect treatment comparisons suggest that maralixibat may provide additional clinical benefit compared to odevixibat in terms of the proportion of serum bile acid responders (estimated treatment difference 32.3%), although head-to-head trials are lacking [114].

**Figure 4:**
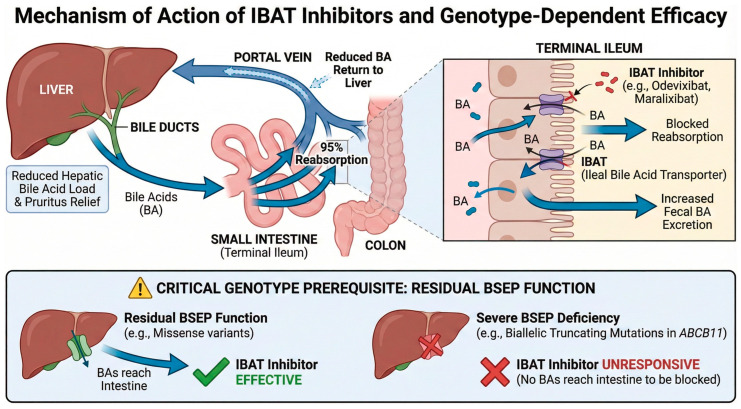
Mechanism of action of IBAT inhibitors and genotype-dependent efficacy. This diagram illustrates the targeted pharmacological interruption of the enterohepatic circulation. (**Top**) Ileal bile acid transporter (IBAT) inhibitors (e.g., odevixibat, maralixibat) block the physiological reabsorption of bile acids in the terminal ileum. This blockade promotes fecal excretion, thereby reducing the toxic bile acid load returning to the liver and alleviating pruritus. (**Bottom**) The efficacy of this approach is strictly genotype-dependent. Because the drug acts in the intestinal lumen, it requires bile acids to be present there. Consequently, patients with residual BSEP function (e.g., missense variants) benefit from treatment as bile acids successfully reach the intestine. In contrast, patients with severe BSEP deficiency (e.g., biallelic truncating mutations in *ABCB11*, corresponding to BSEP3 genotype) are generally unresponsive because the primary canalicular defect prevents bile acids from entering the biliary tree and reaching the site of drug action.

Both drugs act by blocking bile acid reabsorption in the terminal ileum, thereby interrupting enterohepatic circulation and reducing hepatic bile acid load [108,113] (Figure 4). This mechanism requires that bile acids reach the intestinal lumen. Consequently, patients with severe BSEP deficiency (complete absence of functional BSEP protein due to biallelic truncating mutations in *ABCB11*) are generally unresponsive, as the primary defect prevents bile acid secretion into bile. In contrast, patients with residual transporter function (missense variants with partial activity) may derive significant benefit [109,115]. Both agents are generally well tolerated, with the most common adverse events being gastrointestinal (diarrhea, abdominal pain), which are expected given the mechanism of action and are typically mild to moderate in severity [110,116]. Emerging data suggest that IBAT inhibitors may also improve event-free survival and potentially alter disease progression, expanding their role beyond symptomatic relief [117]. Other IBAT inhibitors are currently undergoing clinical trials to evaluate their efficacy in other cholestatic conditions affecting adults or are awaiting final approval, including linerixibat for the treatment of cholestatic pruritus in patients with primary biliary cholangitis (PBC) [118], and volixibat for the management of pruritus in PBC (https://clinicaltrials.gov/study/NCT05050136 (accessed on 20 December 2025)), primary sclerosing cholangitis (PSC) (https://clinicaltrials.gov/study/NCT04663308 (accessed on 20 December 2025)) and intrahepatic cholestasis of pregnancy [102,111].

When medical management fails, surgical intervention is required. Biliary diversion procedures, such as partial biliary diversion (PBD) and ileal bypass, aim to mechanically interrupt the enterohepatic circulation [119,120]. Liver transplantation (LT) remains the definitive therapeutic option for ESLD or refractory pruritus, improving symptoms in 75–100% of patients [121]. Of note, patients progressing to ESLD frequently develop systemic complications beyond hepatic dysfunction, including hepatorenal syndrome—driven by splanchnic vasodilation and renal hypoperfusion—and associated fluid and electrolyte disturbances such as dilutional hyponatremia, hyperkalemia, and refractory ascites, which significantly impact pre-transplant management [122,123]. The choice of LT must consider the genetic defect; for instance, in PFIC1 (*ATP8B1* deficiency), extrahepatic manifestations such as diarrhea may persist or worsen post-transplant due to the systemic expression of the gene.

### 5.2. Management of Specific Conditions

In BRIC, treatment aims to limit the duration of cholestatic episodes. UDCA, rifampicin, and cholestyramine are the mainstays of therapy [124,125,126], while in severe, refractory attacks, invasive procedures such as nasobiliary drainage and albumin dialysis may be considered [127,128].

For ICP, pharmacological treatment primarily relies on UDCA to relieve maternal pruritus and improve liver biochemical profiles. Postpartum follow-up is crucial to confirm the resolution of biochemical abnormalities, validating the diagnosis [38].

In DIC, the cornerstone of management is the immediate withdrawal of the offending agent. Causality assessment is supported by validated scales such as the CIOMS/RUCAM [129] and the RECAM [130].

For LPAC, long-term UDCA therapy (10 mg/kg/day) represents the cornerstone of management, reducing biliary lithogenicity by restoring phospholipid solubilization. Cholecystectomy is indicated for symptomatic gallstones but does not prevent recurrence without concomitant UDCA. Complicated forms (recurrent cholangitis, intrahepatic abscess, massive lithiasis) may require endoscopic, radiological, or surgical intervention [54,131].

## 6. Conclusions

The classification of cholestatic liver diseases has evolved from a rigid, symptom-based nomenclature to a dynamic, molecularly defined continuum. The delineation of 13 distinct PFIC types and their overlap with adult-onset conditions (AOCD) illustrates that pediatric and adult cholestasis are often different facets of the same genetic reality. This conceptual shift—from phenotype-first to genotype-first classification—has profound implications for both clinical practice and research.

The recognition that cholestasis can arise from defects at multiple levels of the bile secretion machinery—canalicular transport (PFIC1–3), tight junction integrity (PFIC4, PFIC7), nuclear receptor signaling (PFIC5), basolateral export (PFIC6), intracellular trafficking (PFIC8–12), and ciliary function (PFIC13)—underscores the remarkable complexity of hepatobiliary homeostasis. Importantly, this expanded molecular nosology has revealed that identical clinical presentations may harbor distinct genetic etiologies, each with specific prognostic and therapeutic implications.

In this new era, genetics is no longer a confirmatory endpoint but the starting point of clinical management. From the early exclusion of biliary atresia to the selection of candidates for IBAT inhibitors, the specific molecular diagnosis drives decision-making at every step. The validation of genotype-based risk stratification models, such as the BSEP1/BSEP2/BSEP3 classification for ABCB11 deficiency, exemplifies how molecular data can now predict native liver survival, hepatocellular carcinoma risk, and response to surgical biliary diversion with clinically actionable precision. These correlations transform genetic testing from a diagnostic tool into a prognostic instrument that directly informs therapeutic choices.

The advent of IBAT inhibitors represents a watershed moment in the management of hereditary cholestasis. For the first time in this specific disease context, a pharmacological class has been designed to target a defined step in the pathophysiological cascade—the enterohepatic circulation of bile acids—offering a mechanism-based intervention that complements the established, albeit broader, cytoprotective effects of UDCA, which has already demonstrated a disease-modifying impact on the natural history of other chronic cholestatic conditions such as primary biliary cholangitis. However, the efficacy of these agents is itself genotype-dependent: patients with residual transporter function stand to benefit most, while those with complete protein absence may require alternative strategies. This pharmacogenomic dimension reinforces the imperative of precise molecular characterization prior to treatment initiation.

Several challenges remain. The interpretation of Variants of Uncertain Significance (VUS) continues to limit diagnostic yield, particularly along the phenotypic continuum where genotype–phenotype correlations are less defined. The periodic re-analysis of genomic data, as recommended by current guidelines, will be essential to reclassify variants as functional evidence accumulates. Furthermore, the contribution of modifier genes and environmental factors to phenotypic variability warrants systematic investigation.

In parallel with genomic advances, recent developments in tissue-based analytical technologies may offer complementary approaches to address these unresolved challenges. In particular, matrix-assisted laser desorption/ionization mass spectrometry imaging (MALDI-MSI) has emerged as a spatially resolved molecular profiling tool capable of mapping the distribution of bile acids, lipids, proteins, and drug metabolites directly within liver tissue sections [132,133,134,135]. Recent studies have demonstrated the ability of MALDI-MSI to visualize the spatial distribution of bile salt species across hepatic parenchyma in cholestatic conditions, identifying molecular structural markers that distinguish healthy from diseased tissue [132], and to characterize the zonation patterns of bile acid metabolism [134]. Moreover, this technology has shown promise in distinguishing mechanisms of biliary toxicity based on the cellular distribution of compounds within the hepatobiliary system [135]. By bridging classical histopathology with unbiased molecular analysis, MALDI-MSI and related spatial metabolomic approaches could, in the future, provide functional tissue-level evidence to support the interpretation of novel VUS and refine genotype–phenotype correlations [133,136]. Nonetheless, it must be emphasized that liver biopsy remains an invasive procedure carrying inherent risks—particularly in pediatric patients—and that the clinical validation, standardization, and accessibility of MALDI-MSI are still under active investigation. Therefore, while these technologies hold considerable potential to enrich the evolving diagnostic approach when biopsy is clinically indicated, they should be considered as a future complementary layer rather than a justification for expanding the indications for invasive tissue sampling.

Looking ahead, the integration of Next-Generation Sequencing with functional validation and emerging biotechnologies—including gene therapy approaches and novel pharmacological targets—promises to transform these complex, often debilitating disorders into manageable, treatable conditions. International collaborative networks, such as the European Reference Network for Rare Liver Diseases (ERN RARE-LIVER) and multicenter consortia like NAPPED, will be instrumental in generating the large-scale genotype–phenotype data necessary to refine prognostic models and optimize therapeutic algorithms.

In conclusion, hereditary cholestatic liver diseases stand at the forefront of precision medicine in hepatology. The molecular redefinition of this disease spectrum has not only deepened our understanding of bile acid biology but has also delivered tangible clinical benefits: earlier diagnosis, accurate prognosis, and targeted therapy. As the field continues to evolve, maintaining rigorous integration of genetic, functional, and clinical data will be essential to fulfill the promise of personalized care for patients across the entire age spectrum.

## Figures and Tables

**Table 1:** Major molecular, biochemical, and clinical characteristics of the 13 identified PFIC types. This table summarizes the expanded nosology of PFIC, correlating specific gene defects with their corresponding protein functions and clinical phenotypes. It highlights the diagnostic utility of serum gamma-glutamyl transferase (GGT) levels in distinguishing between subtypes—contrasting the “normal/low GGT” signature of PFIC1, 2, 4, 5, 7, 10, 11, and 12 with the elevated levels typical of PFIC3, 6, 8, and 9. The classification reflects the diverse pathophysiology of cholestasis, ranging from primary transporter defects to impairments in tight junctions, intracellular trafficking, and nuclear receptor signaling.

PFIC Type	Mutated Gene	Protein Defect	Protein Function	GGT Levels	Hallmark Clinical Features	Age of Onset
PFIC1	*ATP8B1*	FIC1	ATP-dependent flippase translocating phospholipids from the external to the internal membrane leaflet of epithelial cells	Normal/low	Jaundice, severe pruritus, frequent extrahepatic symptoms	First months of life
PFIC2	*ABCB11*	BSEP	Main ABC transporter responsible for the export of bile salts from hepatocytes	Normal/low	Persistent jaundice, severe pruritus, hepatomegaly, rapid progression to end-stage liver disease, increased risk of HCC	First months of life
PFIC3	*ABCB4*	MDR3	ATP-dependent floppase translocating phosphatidylcholine from the internal to the external membrane leaflet of hepatocytes	High	Increased biliary lithogenicity and small bile ducts obstructions	Later infancy/young adulthood
PFIC4	*TJP2*	TJP2/ZO-2	Cytoplasmic protein involved in cell-cell junctional complexes formation by linking cytoskeletal components and integral membrane proteins	Normal/low	Possible extrahepatic symptoms, rapid progression to end-stage liver disease	First days or months of life
PFIC5	*NR1H4*	FXR	Ligand-activated transcription factor which bounds bile acids and regulates the expression of genes involved in bile acid synthesis and transport	Normal/low	Rapid progression to end-stage liver disease, vitamin K-independent coagulopathy	First days or weeks of life
PFIC6	*SLC51A*	OST⍺	Alpha subunit of the OST⍺/OSTß transporter responsible for the efflux in the portal circulation of bile acids reabsorbed from the intestine	High	Failure to thrive, congenital diarrhea	First days of life
PFIC7	*USP53*	USP53	De-ubiquitinating enzyme lacking de-ubiquitinase activity and interacting with the TJP2 protein	Normal/low	Jaundice, severe pruritus, mild and intermittent cholestatic symptoms	From early infancy to adolescence
PFIC8	*KIF12*	KIF12	Microtubule-associated molecular motor involved in intracellular transport of organelles and cytoskeletal organization	High	Jaundice, liver fibrosis, bile ducts proliferation	First weeks of life
PFIC9	*ZFYVE19*	ZFYVE19	Crucial role in the formation of primary cilia in post-mitotic cells	High	Jaundice, hepatosplenomegaly	Infancy
PFIC10	*MYO5B*	MYO5B	Actin-dependent molecular motor involved in plasma membrane recycling systems in polarized and non-polarized cells	Normal/low	Jaundice, pruritus, hepatomegaly, possible intestinal involvement (diarrhea)	Within the first 2 years of life
PFIC11	*SEMA7A*	SEMA7A	Membrane-bound signaling protein contributing to tissue homeostasis and morphogenesis	Normal/low	Jaundice, absence of pruritus	Infancy
PFIC12	*VSP33B*	VSP33B	Involved in intracellular transport and lysosomal function	Normal/low	Jaundice, severe pruritus, occasional hepatosplenomegaly	First weeks of life
PFIC13	*PSKH1*	PSKH1	Still unclear	Unspecified	Hepatorenal ciliopathy characterized by progressive liver dysfunction and progressive chronic renal failure	Infancy

## Data Availability

No new data were created or analyzed in this study. Data sharing is not applicable to this article.

## Abbreviations

The following abbreviations are used in this manuscript:

PFIC	Progressive Familial Intrahepatic Cholestasis
AOCD	Adult-Onset Cholestatic Disease
NGS	Next-Generation Sequencing
IBAT	Ileal bile acid transporter
ETC	Electron transport chain
TPN	Total parenteral nutrition
DILI	Drug-induced liver injury
ESLD	End-stage liver disease
OMIM	Online Mendelian Inheritance in Man
BRIC	Benign Recurrent Intrahepatic Cholestasis
ICP	Intrahepatic Cholestasis of Pregnancy
BSEP	Bile salt export pump
GGT	Gamma-glutamyl transferase
PC	Phosphatidylcholine
DIC	Drug-Induced Cholestasis
LPAC	Low-Phospholipid-Associated Cholelithiasis
ABC	ATP-binding cassette
FIC	Familial Intrahepatic Cholestasis
BA	Biliary atresia
MRCP	Magnetic resonance cholangiopancreatography
WES	Whole Exome Sequencing
WGS	Whole Genome Sequencing
VCTE	Vibration-Controlled Transient Elastography
EASL	European Association for the Study of the Liver
ESPGHAN	European Society for Pediatric Gastroenterology, Hepatology and Nutrition
ALT	Alanine aminotransferase
AST	Aspartate aminotransferase
ALP	Alkaline phosphatase
ULN	Upper limit of normal
ANA	Antinuclear antibodies
AMA	Antimitochondrial antibodies
CT	Computed tomography
CNV	Copy Number Variation
VUS	Variant of Uncertain Significance
ACMG	American College of Medical Genetics and Genomics
NAPPED	NAtural course and Prognosis of PFIC and Effect of biliary Diversion
NLS	Native Liver Survival
SBD	Surgical biliary diversion
HLA	Human leukocyte antigen
UDCA	Ursodeoxycholic acid
MCT	Medium-chain triglyceride
EMA	European Medicines Agency
FDA	Food and Drug Administration
PBC	Primary biliary cholangitis
PSC	Primary sclerosing cholangitis
PBD	Partial biliary diversion
LT	Liver transplantation
CIOMS	Council for International Organizations of Medical Sciences
RUCAM	Roussel Uclaf Causality Assessment Method
RECAM	Revised Electronic Causality Assessment Method
MALDI-MSI	Matrix-assisted laser desorption/ionization mass spectrometry
ERN RARE-LIVER	European Reference Network for Rare Liver Diseases
